# Monitoring of vegetation chlorophyll content in photovoltaic areas using UAV-mounted multispectral imaging

**DOI:** 10.3389/fpls.2025.1643945

**Published:** 2025-08-13

**Authors:** Ming Li, Weiyi Wang, Haoran Li, Zekun Yang, Jianjun Li

**Affiliations:** School of Energy and Transportation Engineering, Inner Mongolia Agricultural University, Hohhot, China

**Keywords:** UAV, chlorophyll content, vegetation index, texture feature, deep learning

## Abstract

The rapid and accurate acquisition of vegetation information, particularly chlorophyll content, is essential for effective vegetation management and ensuring the safe operation of photovoltaic power plants. In this study, the vegetation within a photovoltaic power plant served as the research subject, and multispectral images were acquired using unmanned aerial vehicles, while *in situ* chlorophyll measurements were obtained through ground-based sampling at multiple time points. From these images, twenty vegetation indices and thirty-two texture features were extracted. To reduce feature redundancy and enhance modeling efficiency, feature selection was performed using the minimum redundancy maximum relevance method and Pearson correlation analysis. The selected features were then used in three modeling strategies—vegetation index–based, texture feature–based, and fused index–texture–based—employing three conventional machine-learning regressors (partial least squares regression, random forest, support vector machine regression) and three deep-learning regressors (back propagation neural network, convolutional neural network, multilayer perceptron). Based on the optimal model, a spatiotemporal distribution map of chlorophyll content within the study area was generated. The results indicated that both vegetation indices and texture features exhibited significant correlations with chlorophyll content, with the strongest correlation observed between the green normalized difference vegetation index (GNDVI) and the NIR_Mean (Pearson coefficients of 0.82 and 0.65, respectively). Moreover, the fusion of vegetation indices and texture features effectively improved the accuracy of chlorophyll inversion models; among the six regression algorithms tested, the multilayer perceptron model achieved the highest performance (R² = 0.874, RMSE = 3.725, MAPE = 3.982%). This study provides a novel method for monitoring chlorophyll content in vegetation within photovoltaic power plant regions and offers informational support for refined regional vegetation management.

## 1 Introduction

Vegetation plays a crucial role in the integration of photovoltaic systems and sand control. The cultivation of crops such as purple locust, scutellaria, and chicory beneath and between photovoltaic panels not only enhances soil quality but also generates significant economic benefits. Effective and efficient vegetation management can reduce dust accumulation on photovoltaic panels, thereby enhancing power generation efficiency, minimizing the risk of fire and other hazards, and ensuring the safe and stable operation of photovoltaic power plants (Vaverková et al., 2022). Chlorophyll, a vital pigment in plant photosynthesis, serves as an indicator of plant health and growth, contributing significantly to biomass accumulation. Its content has been widely utilized for monitoring vegetation growth and diagnosing plant diseases (Wu et al., 2023). Traditional chlorophyll detection methods typically involve measuring leaf absorbance at specific wavelengths using spectrophotometers, followed by content estimation through empirical formulas. These methods are destructive, time-consuming, and labor-intensive, making them unsuitable for large-scale, real-time monitoring. Although handheld chlorophyll meters provide rapid, non-destructive, real-time field measurements (Uddling et al., 2007), their single-point readings are often affected by environmental variables such as leaf condition, structure, and ambient light, limiting their applicability in large-scale vegetation management within photovoltaic power plant areas.

Chlorophyll content significantly influences plant reflectance in the visible and near-infrared regions, thereby establishing a strong correlation with spectral data (Neto et al., 2017). The rapid monitoring of vegetation growth using multispectral sensors on remote sensing satellites has been extensively applied (Gong et al., 2024). However, the spatial resolution of commonly used satellite imagery is typically around 10 meters, and image quality is susceptible to atmospheric interference, limiting its effectiveness in fine-scale vegetation monitoring. Recently, the rapid development of unmanned aerial vehicle (UAV) technology has led to a significant reduction in remote sensing costs. UAVs equipped with high-definition cameras can achieve centimeter-level resolution and provide more accurate monitoring. Additionally, UAV remote sensing is not constrained by orbital cycles or revisit frequency and can capture real-time vegetation growth status with high precision (de Castro et al., 2021). Compared to UAV-based hyperspectral cameras, multispectral cameras capture image data reflecting electromagnetic radiation only across a limited number of discrete spectral bands, offering advantages such as lower cost, easier data acquisition, and reduced computational complexity (Adão et al., 2017). They also help avoid the “curse of dimensionality” associated with hyperspectral data, making them a cost-effective and efficient solution for vegetation monitoring (Lu et al., 2019). For instance, Haghighattalab et al. used UAV-mounted RGB and multispectral cameras to develop a semi-automated method for extracting wheat phenotypic traits (Haghighattalab et al., 2016). Liebisch et al. combined thermal imaging with multispectral data to differentiate maize genotypes based on canopy cover and vegetation indices (Liebisch et al., 2015). Duan et al. compared UAV-derived NDVI at various wheat growth stages with data from a handheld active NDVI sensor, demonstrating that NDVI at the flowering stage was more strongly correlated with yield after adjusting for ground cover (Duan et al., 2017).

Compared with traditional regression approaches such as linear regression, machine learning-based regression algorithms offer greater generalization capability and are better suited for processing large datasets and modeling nonlinear relationships. These algorithms can also mitigate overfitting through techniques such as regularization and early stopping. Wu et al. employed four machine learning algorithms—deep neural network, partial least squares, random forest (RF), and AdaBoost—to construct models for estimating the chlorophyll content of wheat at various growth stages based on 26 selected vegetation indices and measured chlorophyll data (Wu et al., 2023). Hawryło et al. calculated 23 vegetation indices, including the green normalized difference vegetation index, from satellite-based multispectral remote sensing data, and used three machine learning algorithms—k-nearest neighbors, RF, and support vector machine—to model defoliation in pine forests in western Poland (Hawryło et al., 2018). These studies demonstrate the effectiveness of combining multispectral remote sensing and machine learning for crop monitoring. However, their application in vegetation management for photovoltaic power plants remains relatively unexplored. We hope this study will contribute to the existing body of literature on chlorophyll content estimation, particularly in hard-to-access areas such as photovoltaic power stations, given the growing accessibility and efficiency of UAV-based multispectral surveys.

Rapid and accurate vegetation growth monitoring is essential for effective vegetation management and the safe operation of photovoltaic power facilities. In this study, vegetation within a photovoltaic power plant was selected as the research target. Multispectral images were acquired using UAVs, while *in situ* chlorophyll measurements were obtained through ground-based sampling at multiple time points. Partial least squares regression (PLSR), random forest, support vector machine regression (SVR), back propagation neural networks (BPNN), convolutional neural networks (CNN), and multilayer perceptrons (MLP) were employed to construct models for estimating chlorophyll content based on vegetation indices and texture features. The minimum redundancy maximum relevance (mRMR) method and Pearson correlation analysis were used to select features most relevant to chlorophyll content. The performance of different modeling algorithms was then compared under identical feature sets.

Therefore, the overall objective of this study is to develop an accurate and efficient method for estimating vegetation chlorophyll content in photovoltaic power plant areas using UAV multispectral imagery. Specifically, the study aims to: (1) extract and evaluate vegetation indices and texture features relevant to chlorophyll content; (2) apply feature selection techniques to identify the most informative predictors; and (3) construct and compare multiple regression and deep learning models to determine the most effective approach for chlorophyll estimation.

## 2 Materials and methods

### 2.1 Description of the study area and field data acquisition

**Figure 1:**
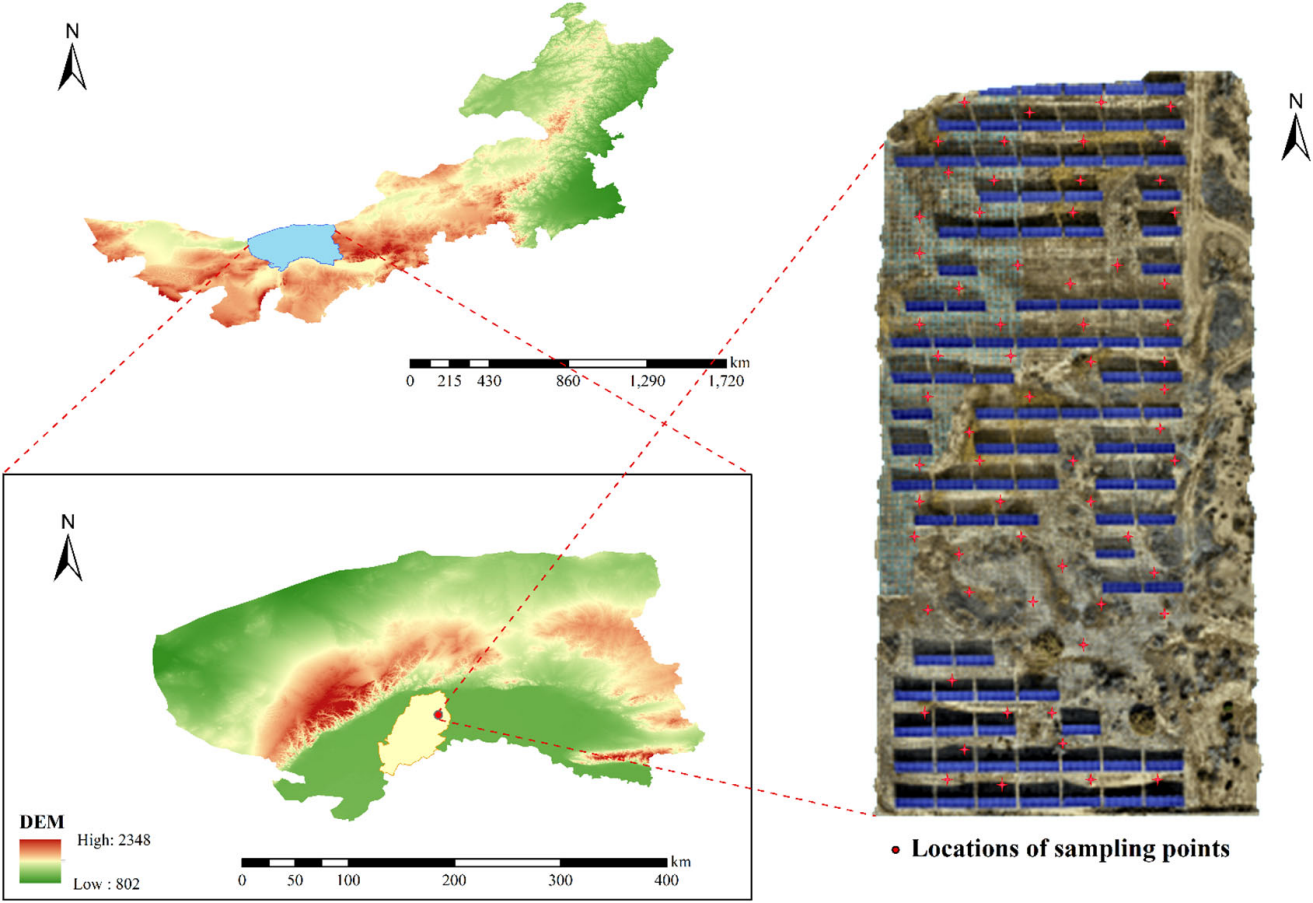
Study area map.

The study area is located within a photovoltaic power station in Linhe District, Bayannur City, Inner Mongolia Autonomous Region (41°02′22.4570″N, 107°35′39.4411″E), encompassing an experimental area measuring 150 meters in width and 300 meters in length. This region features a temperate continental climate characterized by four distinct seasons, with hot and rainy summers and cold, dry winters. The average annual temperature ranges between 8–10 °C, with annual precipitation of approximately 200–300 mm and total annual sunshine duration ranging from 3000 to 3200 hours. Ground-based chlorophyll measurements were conducted on September 15, October 15, and December 15, 2024, at 64 sampling points evenly distributed across the study area using a SPAD502 portable chlorophyll meter (**Figure 1**). Due to reduced vegetation growth during the winter months, sampling frequency was accordingly decreased to focus data collection on periods with more pronounced physiological activity. The geographic coordinates of each sampling point were recorded to enable subsequent overlay display and spatial analysis in conjunction with UAV multispectral imagery. The vegetation type at each sampling point was identified, and three canopy leaves were selected from each plant. Each leaf was measured three times, and the average of all nine readings was calculated to determine the chlorophyll content of the sample. As shown in **Table 1**, a total of 2,691 valid chlorophyll measurements from ground vegetation were obtained for four crop species: purple locust (*Robinia pseudoacacia*), chicory (*Cichorium intybus*), reed (*Phragmites australis*), and scutellaria (*Scutellaria baicalensis*). These field observations of chlorophyll content were used both to train the models and to serve as ground truth for validation. To enhance the model’s robustness and generalization ability, all field measurements from the three sampling periods were combined into a unified dataset, increasing the sample size and capturing temporal variability in vegetation conditions to improve model performance under varying environmental and seasonal conditions.

**Table 1:** Chlorophyll content measurements and statistical analyses in vegetation.

Crop species	Sample size	Maximum	Minimum	Average	Standard deviation
purple locust	613	40.4	29.8	36.78	4.06
chicory	526	40.2	27.4	34.07	4.32
reed	959	65.2	43.1	51.19	4.41
scutellaria	593	46.3	32.2	40.56	3.04

### 2.2 Multispectral data acquisition and preprocessing

A DJI Mavic 3M (multispectral version) drone with real-time kinematic (RTK) capability was employed to acquire vegetation multispectral imagery concurrently with ground-based vegetation sampling. RTK corrections were obtained via a network RTK service, without the use of a physical base station. The drone supports a maximum payload of 1.05 kg, operates in wind speeds up to 12 m/s, and functions within ambient temperatures ranging from -10°C to 40°C. The onboard multispectral camera includes a visible sensor and four spectral bands: green (560 ± 16 nm), red (650 ± 16 nm), red edge (730 ± 16 nm), and near-infrared (860 ± 26 nm). Although the UAV system also captured visible images, these data were not used in this study, as the analysis focused exclusively on information derived from a multispectral imaging sensor. Data acquisition was conducted between 12:00 and 14:00 to minimize variations in solar illumination and shadow interference. Prior to each flight, radiometric calibration plates with 25% and 50% reflectance were photographed, and their positions were recorded to support subsequent radiometric and geometric corrections. A single-grid flight mission was used, with parallel flight lines automatically generated to ensure complete coverage of the study area. The flight was conducted at an altitude of 25 m and a speed of 5 m/s, with a shooting interval of 2 s and 85% forward overlap and 85% side overlap. This flight configuration allowed for the acquisition of high-quality, continuous nadir imagery suitable for vegetation index extraction and orthomosaic generation.

Multispectral images were radiometrically corrected and mosaicked using DJI Terra software. The resulting output consisted of single-band orthomosaic images for the green, red, red-edge, and near-infrared bands, each with a ground sampling distance of 0.657 cm/pixel. All the sampling points were delineated as regions of interest based on the geolocation data collected during field sampling, and reflectance values from the four corresponding single-band images were extracted at each sampling point.

### 2.3 Calculation of multispectral vegetation indices and extraction of texture features

#### 2.3.1 Calculation of multispectral vegetation indices

Vegetation indices are widely used indicators derived from multispectral remote sensing data to assess vegetation coverage, health status, and growth dynamics. In this study, 20 vegetation indices highly correlated with chlorophyll content were selected based on the spectral reflectance characteristics of chlorophyll and previous studies conducted both domestically and internationally. The bands used include red (R), green (G), red edge (RE), and near-infrared (NIR). The specific formulas for these indices are listed in **Table 2**.

**Table 2:** Vegetation index name and formula.

Vegetation index	Formula	Brief description	References
CIre	NIR/RE−1	Estimates chlorophyll using red-edge and NIR.	(Gitelson et al., 2005)
DVI	NIR−RE	Simple indicator based on NIR–red-edge difference.	(Yan et al., 2021)
GNDVI	(NIR−G)/(N+G)	Sensitive to chlorophyll via Green and NIR.	(Wang et al., 2007)
MSR	(NIR/R−1)/(sqrt(NIR/R)+1)	Reduces background noise in vegetation detection.	(Xie et al., 2018)
NDVI	(NIR−R)/(NIR+R)	Measures vegetation vigor and biomass.	(Gitelson and Merzlyak, 1994)
NDVIre	(NIR−RE)/(NIR+RE)	NDVI variant using red-edge, more sensitive to chlorophyll.	(Barnes et al., 2000)
NDWI	(G−NIR)/(G+NIR)	Indicates leaf and canopy water content.	(Gao, 1996)
RTVI	100*(NIR−RE)−10*(NIR−G)	Improves linearity in biomass estimation.	(Chen et al., 2010)
RVI	NIR/R	Simple NIR/Red ratio for vegetation density.	(Gonenc and Ozerdem, 2019)
TCARI	3*(NIR−R−0.2*(NIR−G)*(NIR/R))	Reduces non-chlorophyll effects in chlorophyll estimation.	(Haboudane et al., 2002)
WDRI	(0.2*NIR−R)/(0.2*NIR+R)	Handles wide range of vegetation densities.	(Tang et al., 2020)
GRVI	NIR/G	Reflects plant health via Green and NIR bands.	(Motohka et al., 2010)
LCI	(NIR−RE)/(NIR+R)	Estimates chlorophyll using red-edge and NIR.	(Nunez et al., 2013)
CRI	(1/G)−(1/R)	Detects carotenoid pigments related to stress.	(Peng et al., 2018)
Datt2	NIR/RE	Empirical red-edge index for chlorophyll estimation.	(Datt, 1999)
IPVI	NIR/(NIR+R)	Normalized NDVI variant.	(Rondeaux et al., 1996)
GDVI	NIR−G	Early stress detection using Green and NIR.	(Daughtry et al., 1992)
GSAVI	1.5*(NIR−G)/(NIR+G+0.5)	Soil-adjusted green-based NDVI.	(Sripada et al., 2006)
GOSAVI	1.16*(NIR−G)/(NIR+G+0.16)	Optimized soil correction using green band.	(Rondeaux et al., 1996)
GRDVI	(NIR−G)/sqrt(NIR+G)	Sensitive to chlorophyll via Green–NIR difference.	(Roujean and Breon, 1995)

#### 2.3.2 Extraction of texture features

Texture Features are important features used in image processing to describe the image surface structure, spatial patterns, and local grayscale variations. These features reflect detailed information such as repetitive structures, directional tendencies, roughness, and smoothness of object surfaces. In this study, texture features were extracted from the red, green, red-edge, and near-infrared bands using the gray-level co-occurrence matrix (GLCM). The extraction was implemented in Python 3.7 using the scikit-image library, which offers a flexible and efficient framework for GLCM-based texture analysis. During processing, image borders were symmetrically extended to ensure that edge pixels were included in the GLCM computation, thereby retaining texture information across the entire image extent. For each band, eight texture metrics were calculated—mean, variance, homogeneity, contrast, dissimilarity, entropy, second moment, and correlation—resulting in a total of 32 texture features extracted from four spectral bands. The extraction parameters were set as follows: a window size of 8 × 8 pixels, a direction of 90°, a gray level range of 0–255, and a sliding step size of 2. The selected window size allowed for capturing fine-scale texture features while ensuring sufficient statistical variability within each window for reliable computation.

### 2.4 Construction of vegetation chlorophyll estimation models

#### 2.4.1 Feature selection

Feature selection was performed using a combination of the mRMR algorithm and Pearson correlation analysis. First, the mRMR algorithm was applied to rank all candidate vegetation indices and texture features based on their mutual information with chlorophyll content and their redundancy with each other. This step aimed to identify variables with high information gain and low redundancy. Subsequently, Pearson correlation analysis was used to assess the linear relationships between the top-ranked features and measured chlorophyll content. Only features showing statistically significant correlations (p < 0.05) were retained for modeling. This process ensured that the final selected features were not only informative and minimally redundant but also closely associated with the target variable.

##### 2.4.1.1 Pearson correlation analysis

The Pearson Correlation Coefficient is a statistical metric used to quantify the linear relationship between two variables and is commonly applied to assess their degree of dependence. In this study, the PCC matrix was computed using Python, based on the following formula:


$$r=\frac{\sum_{i=1}^{n}(X_{i}-\bar{X})(Y_{i}-\bar{Y})}{\sqrt{\sum_{i=1}^{n}{\left(X_{i}-\bar{X}\right)}^{2}\sum_{i=1}^{n}{\left(Y_{i}-\bar{Y}\right)}^{2}}}$$

In this formula, $X_{i}$ ​ and $Y_{i}$ ​ represent the individual observations in the sample. $\bar{X}$ and $\bar{Y}$ denote the means of variables *X* and *Y*, respectively. The symbol *n* indicates the sample size.

##### 2.4.1.2 Minimum redundancy maximum relevance feature selection

mRMR is a feature selection method used to extract feature subsets from high-dimensional data that exhibit high relevance and low redundancy, thereby enhancing the performance of machine learning models. By utilizing mutual information, mRMR evaluates both relevance and redundancy among features, capturing linear and nonlinear correlations simultaneously. This reduces the impact of feature redundancy, alleviates the risk of dimensionality-related issues, and helps prevent model overfitting. In this study, 10 vegetation indices were selected for models based solely on vegetation indices, 11 texture features for models based solely on texture features, and 21 combined features for models integrating both vegetation indices and texture features to construct the chlorophyll content inversion models.

#### 2.4.2 Construction of machine learning models

For the purpose of evaluating model performance, the entire dataset, consisting of vegetation indices, texture features, and corresponding *in-situ* chlorophyll measurements, was randomly divided into training and validation subsets at a ratio of 3:1. The training set was used to fit the model, while the validation set was used to assess its generalization performance. To investigate the effects of vegetation index, texture features, and their combination on the accuracy of chlorophyll content estimation, we considered the following three machine learning modes.

##### 2.4.2.1 Partial least squares regression

PLSR is a widely used method for modeling high-dimensional data, integrating the strengths of Principal Component Analysis, Multiple Linear Regression, and Canonical Correlation Analysis. PLSR enhances model robustness and predictive accuracy by projecting both independent and dependent variables into a lower-dimensional space in which the projections exhibit maximal correlation. When independent variables are highly correlated or exhibit multicollinearity, PLSR maintains strong performance while effectively reducing dimensionality, minimizing data redundancy, and enhancing computational efficiency. In this study, PLSR was employed for modeling. The number of principal components was set to three, and all other parameters were maintained at their default values to ensure model stability.

##### 2.4.2.2 Random forest regression

RF is a non-parametric ensemble learning method composed of multiple decision trees. Each tree is trained using randomly selected features and bootstrapped samples from the original dataset. RF aggregates the predictions of multiple weak learners through majority voting (for classification) or averaging (for regression). Compared to a single decision tree, RF effectively reduces overfitting and demonstrates robustness to outliers and noise. Additionally, RF enables feature importance assessment, which facilitates feature selection and model optimization. In this study, RF was implemented with 100 decision trees, while other hyperparameters were retained at their default values to maintain computational efficiency.

##### 2.4.2.3 Support vector regression

SVR is widely applied to nonlinear regression tasks due to its robustness and generalization ability. SVR maps data into a high-dimensional feature space via kernel functions and constructs an optimal hyperplane to minimize prediction error. By employing an ϵ-insensitive loss function, SVR calculates errors only for data points that deviate beyond the threshold ϵ, thereby enhancing model robustness. SVR is suitable for small-sample, high-dimensional, and nonlinear datasets. In this study, the radial basis function kernel was employed, with the penalty parameter C set to 4.0, the ϵ-insensitive margin set to 0.03, and the kernel parameter γ set to 1/n, where n is the number of features, to optimize model performance.

#### 2.4.3 Construction of deep learning models

To investigate the effects of vegetation index, texture features, and their combination on the accuracy of chlorophyll content estimation, we considered the following three deep learning modes. The dataset was split into training and validation subsets at a ratio of 3:1, consistent with the partitioning used in the machine learning models.

##### 2.4.3.1 Back propagation neural network

BPNN is an artificial neural network that utilizes the error back propagation algorithm for regression and classification tasks. The BPNN consists of an input layer, one or more hidden layers, and an output layer. Outputs are computed through forward propagation, while network parameters are updated via the gradient descent method. BPNNs effectively learn nonlinear mappings and are suitable for modeling high-dimensional data. In this study, a four-layer BPNN was employed, with two hidden layers consisting of 64 and 32 neurons respectively. The ReLU activation function was applied, Adam optimizer was used with a learning rate of 0.01, and training was conducted for a maximum of 1,000 epochs to ensure model convergence.

##### 2.4.3.2 One-dimensional convolutional neural network

**Figure 2:**
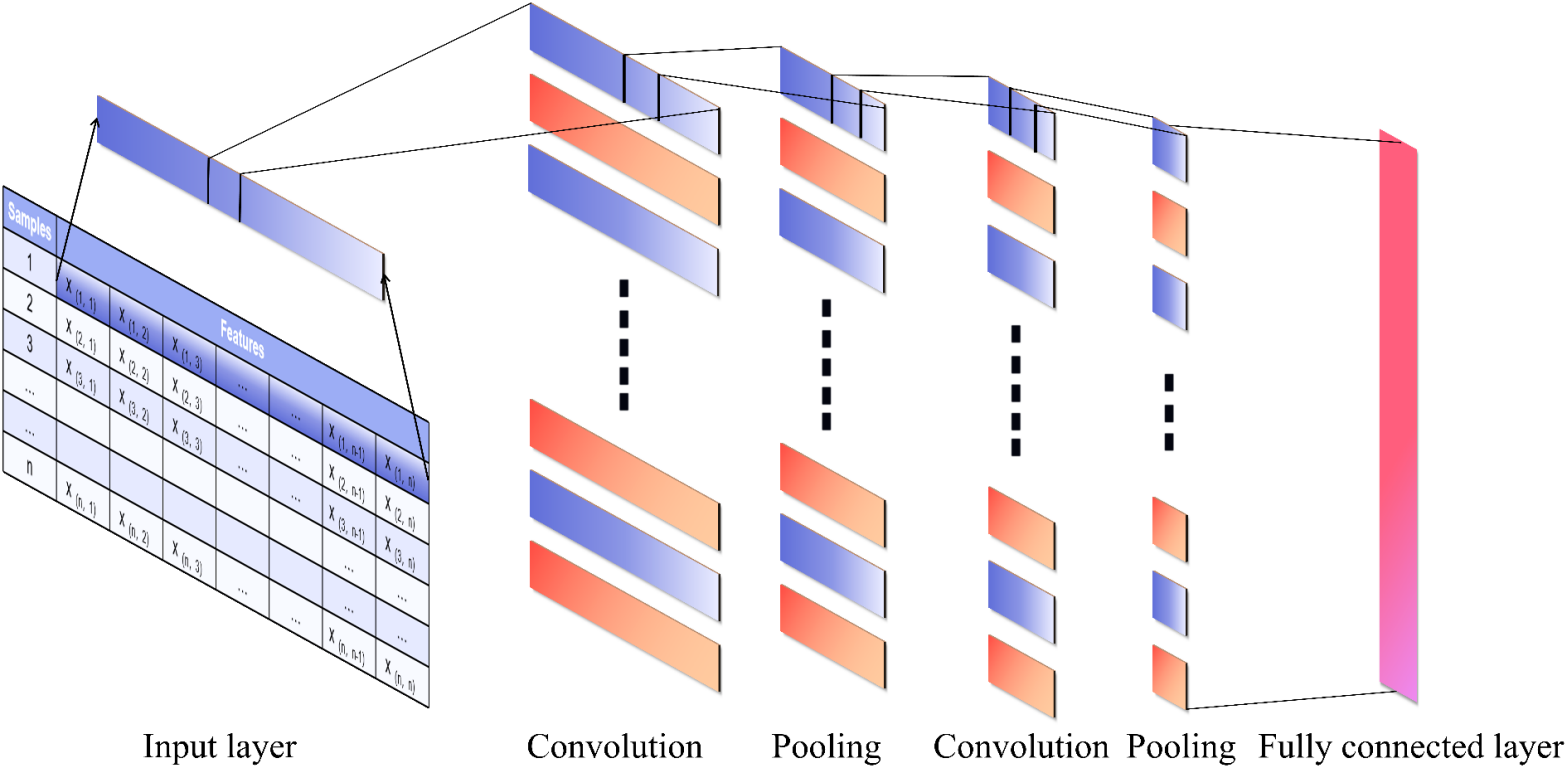
Architecture of the convolutional neural network model.

1D-CNN is primarily applied to the processing of time-series and spectral data due to its ability to extract local patterns within ordered sequences. In this study, although the vegetation indices and texture features were derived from discrete multispectral bands and three separate acquisition dates, they still exhibit an inherent sequential structure, which allows the model to extract local patterns through one-dimensional convolution. The 1D-CNN model leverages local receptive fields and a weight-sharing mechanism, and performs dimensionality reduction via pooling layers to enhance computational efficiency and generalization. Compared with traditional methods, 1D-CNNs can automatically learn key patterns in data and exhibit strong resistance to noise. In this study, a two-layer convolutional network was constructed, with 64 and 32 convolutional kernels (kernel size = 3) in each layer. The ReLU activation function was used, the Adam optimizer was selected with a learning rate of 0.01, and the model was trained for a maximum of 1,000 epochs (**Figure 2**).

##### 2.4.3.3 Multilayer perceptron

MLP is a feed-forward neural network designed to learn complex nonlinear mappings by extracting higher-order features through fully connected layers. Model performance is improved by minimizing the error during training. In this study, an MLP network comprising three hidden layers was constructed, with 64, 32, and 16 neurons in the respective layers. The ReLU function was used as the activation function, Adam was selected as the optimizer, and the learning rate was set to 0.01, while other parameters remained at their default values.

### 2.5 Evaluation metrics for model performance

In this study, the coefficient of determination (R²), root mean squared error (RMSE), and mean absolute percentage error (MAPE) were employed to evaluate 18 models across three modeling strategies (vegetation index-based, texture feature-based, and fused index–texture-based) and six model types. R² measures the goodness of fit, reflecting the correlation between predicted and observed values; values closer to 1 indicate stronger predictive performance. RMSE quantifies the average deviation between predicted and actual values; lower RMSE values indicate higher predictive accuracy. MAPE assesses model performance in percentage terms; lower MAPE values correspond to greater predictive accuracy. The formulas are as follows:


$$R^{2}=1-\frac{\sum_{i=1}^{n}{\left(y_{i}-\hat{y_{i}}\right)}^{2}}{\sum_{i=1}^{n}{\left(y_{i}-\bar{y}\right)}^{2}}$$

$$\text{RMSE}=\sqrt{\frac{1}{\text{n}}{\sum_{\text{i}=1}^{\text{n}}\left({\text{y}}_{\text{i}}-\hat{{\text{y}}_{\text{i}}}\right)}^{2}}$$

$$\text{MAPE}=\frac{1}{\text{n}}\sum_{\text{i}=1}^{\text{n}}|\frac{{\text{y}}_{\text{i}}-\hat{{\text{y}}_{\text{i}}}}{{\text{y}}_{\text{i}}}|\times 100\%$$

In the equations, $y_{i}$ ​ denotes the actual value, $\hat{y_{i}}$ ​ represents the predicted value, $\bar{y}$ ​ is the mean of the actual values, and *n* refers to the total number of samples.

## 3 Results

### 3.1 Vegetation indices and texture features associated with chlorophyll content

**Figure 3:**
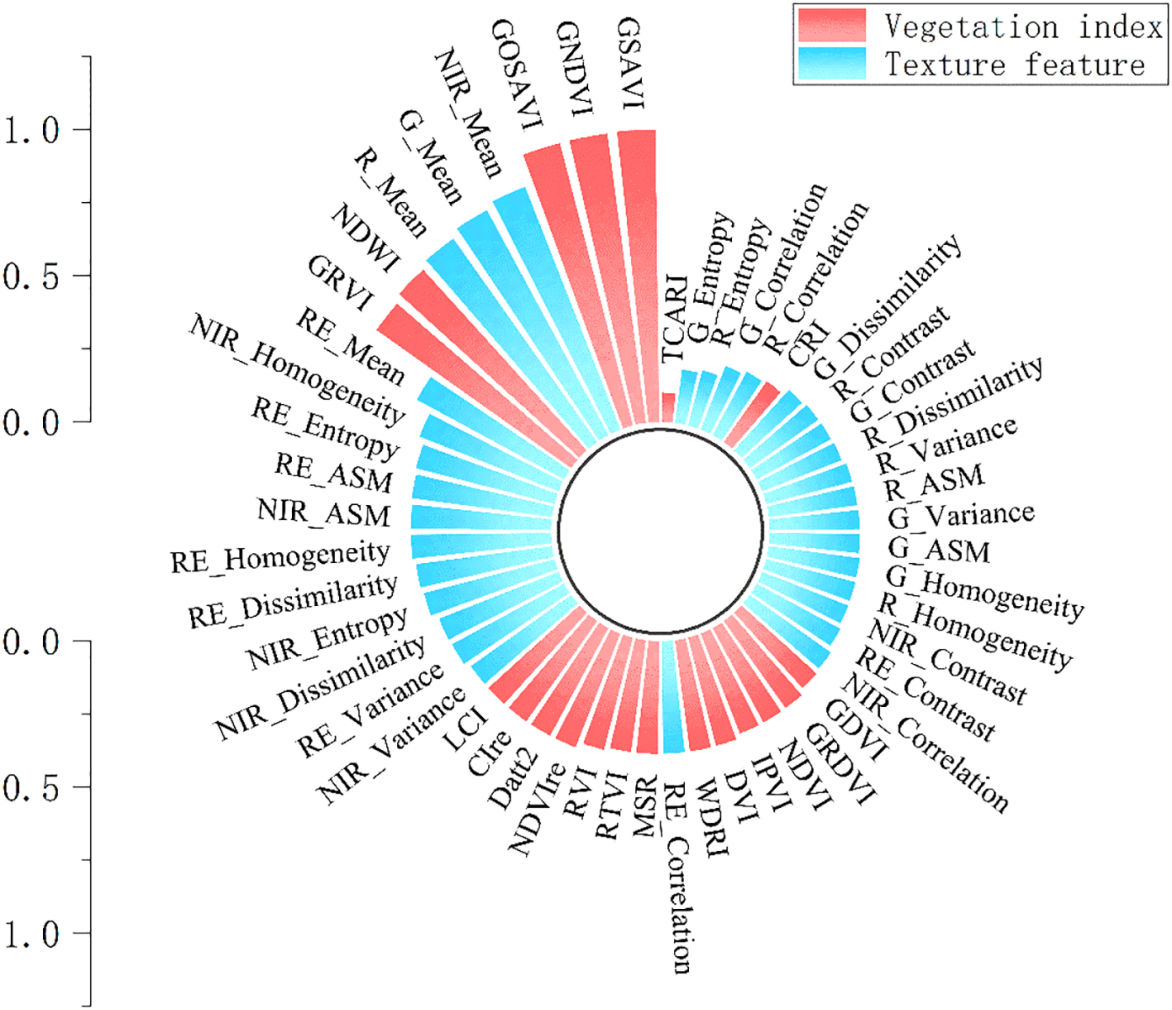
Ranking of feature importances.

To ensure the relevance of feature variables to the regression task while reducing feature redundancy, preventing model overfitting, and enhancing model generalization, the mRMR algorithm was employed to downscale the vegetation indices (Section 2.3.1) and texture features (Section 2.3.2). As shown in **Figure 3**, GSAVI demonstrated the highest relevance with the lowest redundancy among the 20 vegetation indices. Among the 32 texture features extracted from four spectral bands, NIR_Mean exhibited the highest relevance and lowest redundancy.

**Figure 4:**
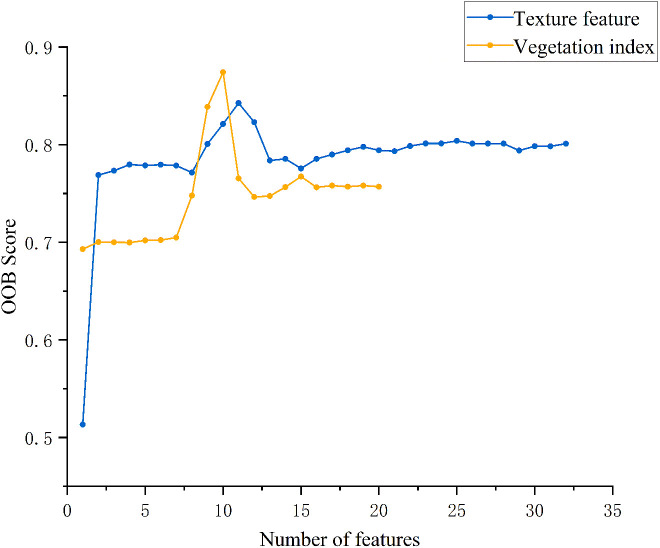
Impact of the number of features on OOB score.

To determine the optimal number of features and mitigate dimensionality issues and overfitting, vegetation index and texture feature models were evaluated using Out-of-Bag (OOB) data. As shown in **Figure 4**, the OOB score for vegetation indices increases steadily as the number of features increases from 1 to 10, peaking at 0.87, after which it shows a slight decline with minor fluctuations. For texture features, the OOB score gradually rises from 1 to 4 features, then slightly decreases before increasing again to a maximum of 0.85 at 11 features, followed by an overall downward trend.

**Figure 5:**
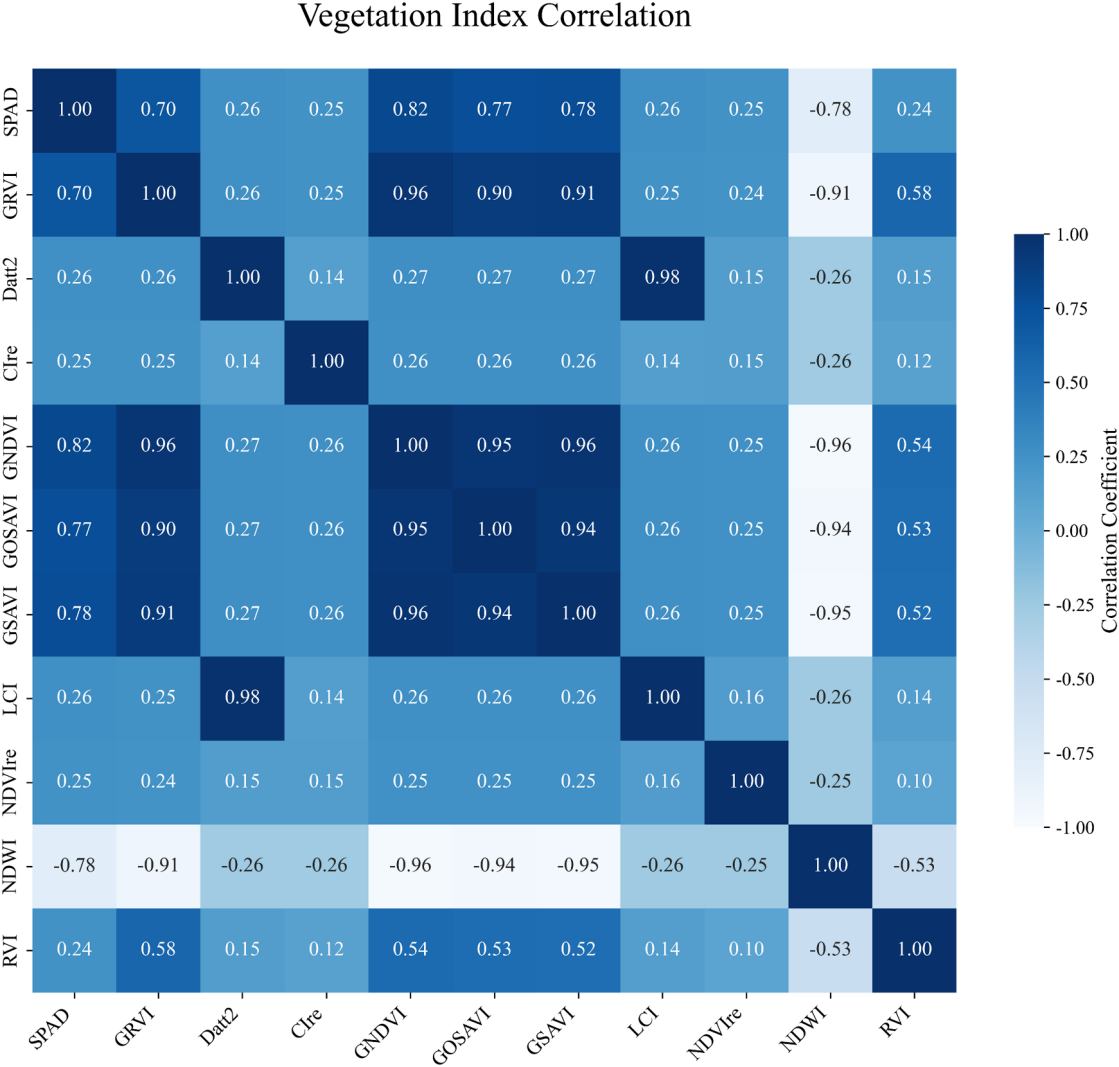
Pearson correlation between vegetation indices and SPAD values.SPAD: A unitless index derived from SPAD meter readings, commonly used to estimate the relative chlorophyll concentration in plant leaves.

**Figure 6:**
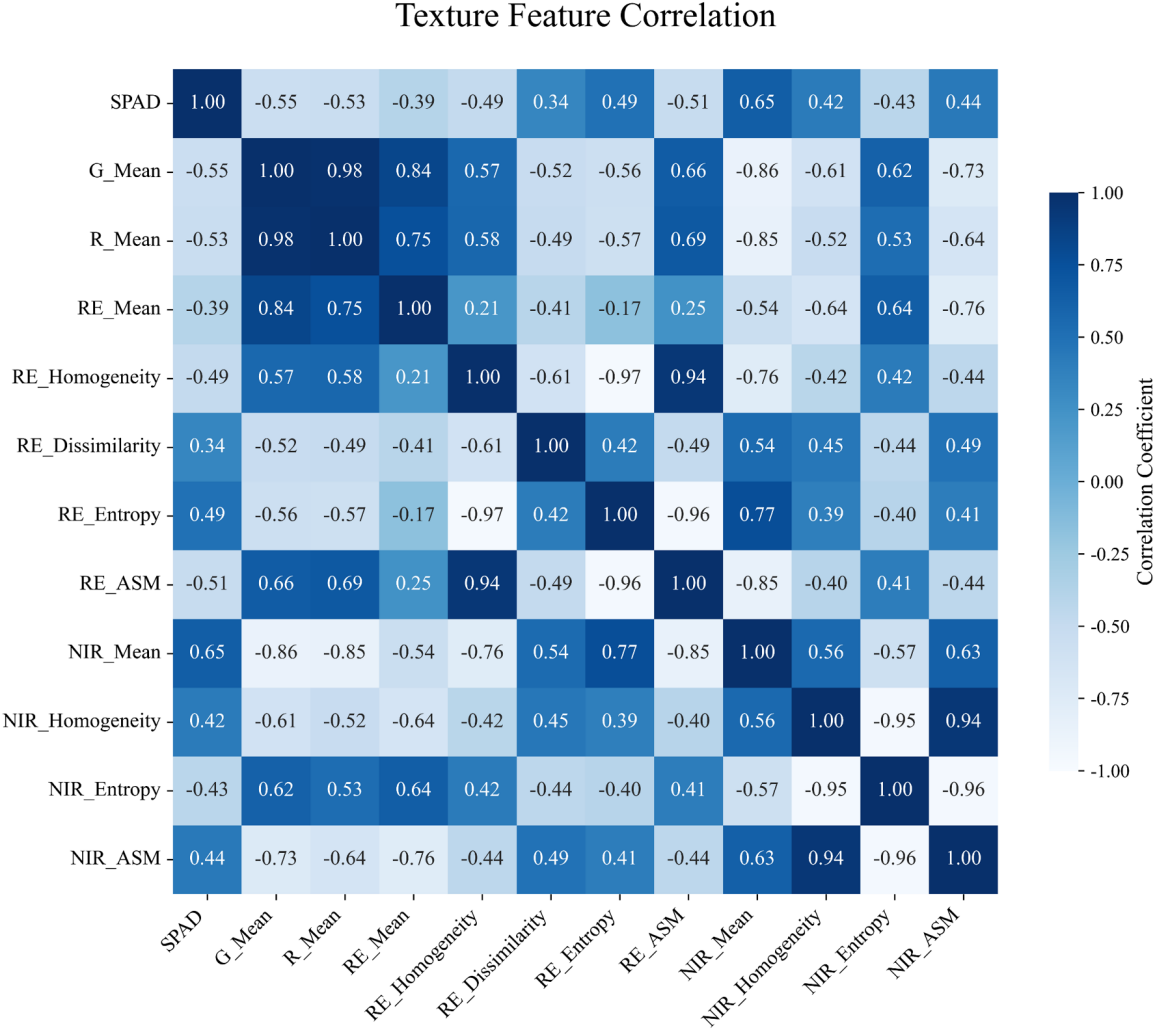
Pearson correlation between texture features and SPAD values.SPAD: A unitless index derived from SPAD meter readings, commonly used to estimate the relative chlorophyll concentration in plant leaves.

Therefore, in this study, 10 vegetation indices with the highest mRMR scores and 11 texture features were selected as input variables for chlorophyll content modeling. Pearson correlation analysis was conducted to assess the significance of these features, as shown in **Figure 5**. All vegetation indices used for model construction were found to be highly significantly correlated with chlorophyll content at the p < 0.001 level. Most indices showed positive correlations, while NDWI exhibited a significant negative correlation. The absolute values of the Pearson correlation coefficients ranged from 0.24 to 0.82, with the highest observed for GNDVI (r = 0.82) and the lowest for RVI (r = 0.24). Similarly, as shown in **Figure 6**, all 11 selected texture features also showed highly significant correlations with chlorophyll content (p < 0.001), with Pearson correlation coefficients ranging from –0.55 to 0.65. The strongest correlation was observed for NIR_Mean (r = 0.65), whereas the weakest was for RE_Dissimilarity (r = 0.34).

### 3.2 Inverse estimation of chlorophyll content based on vegetation indices and texture features

To investigate the effects of vegetation index, texture features, and their combination on the accuracy of chlorophyll content estimation, six modeling algorithms were applied: PLSR, RF, SVR, BP, CNN, and MLP. Each algorithm was trained under three feature input strategies—vegetation index only, texture features only, and a combination of both—with chlorophyll content as the dependent variable.

**Figure 7:**
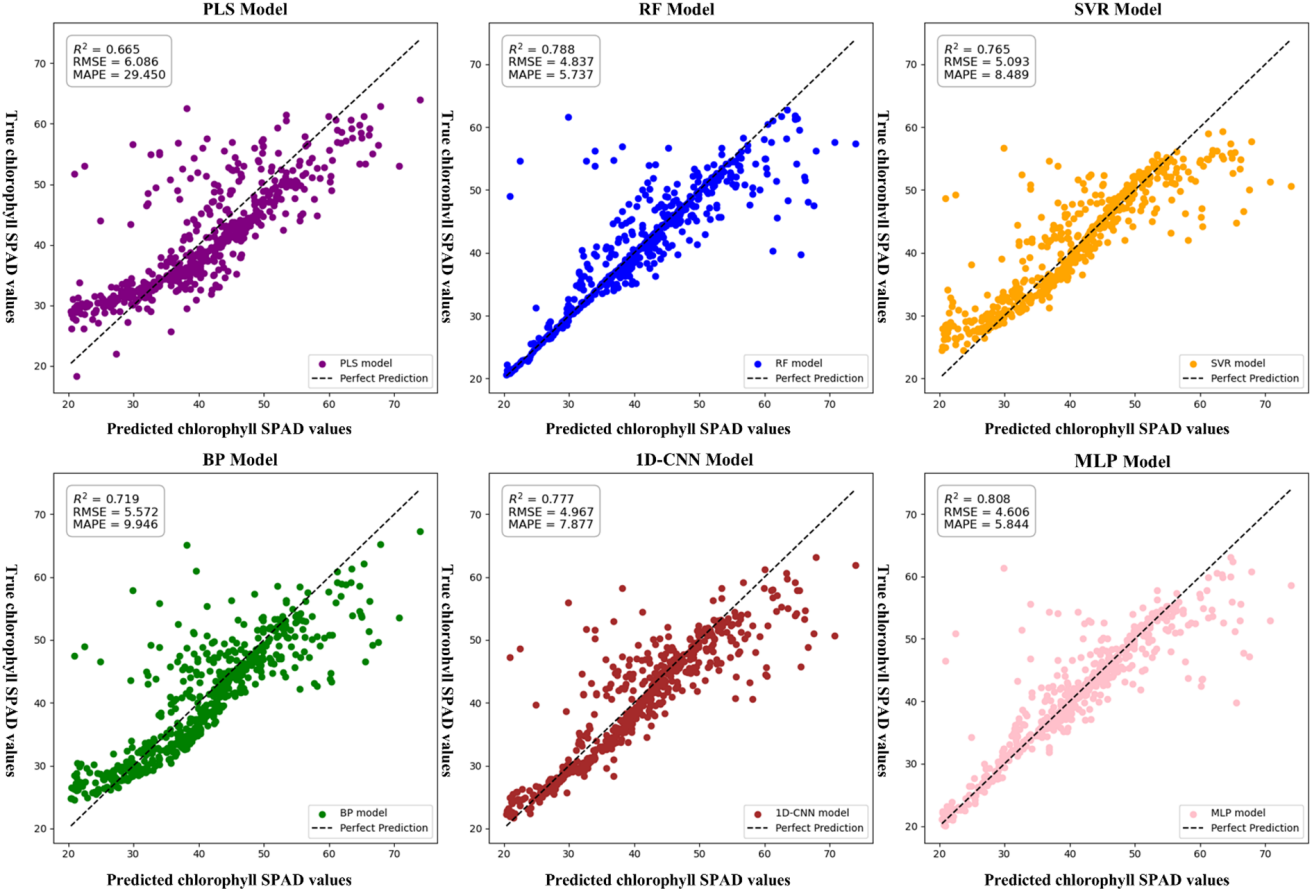
Chlorophyll estimation model based on vegetation indices only.SPAD: A unitless index derived from SPAD meter readings, commonly used to estimate the relative chlorophyll concentration in plant leaves.

In the vegetation index–only models, all algorithms achieved an R² greater than 0.65. The performance ranking was MLP > RF > CNN > SVR > BP > PLSR. The MLP model showed the best performance, with an R² of 0.808, RMSE of 4.606, and MAPE of 5.844. Compared to PLSR, MLP improved R² by 21.51% and reduced RMSE by 32.1%. Compared to RF, SVR, BP, and CNN, R² increased by 2.54%, 5.62%, 12.38%, and 3.99%, and RMSE decreased by 5.02%, 10.57%, 20.97%, and 7.84%, respectively. Although RF performed slightly better than MLP in terms of MAPE, showing a 1.87% improvement—MLP still provided the highest overall accuracy. These comparative results are summarized in **Figure 7**.

**Figure 8:**
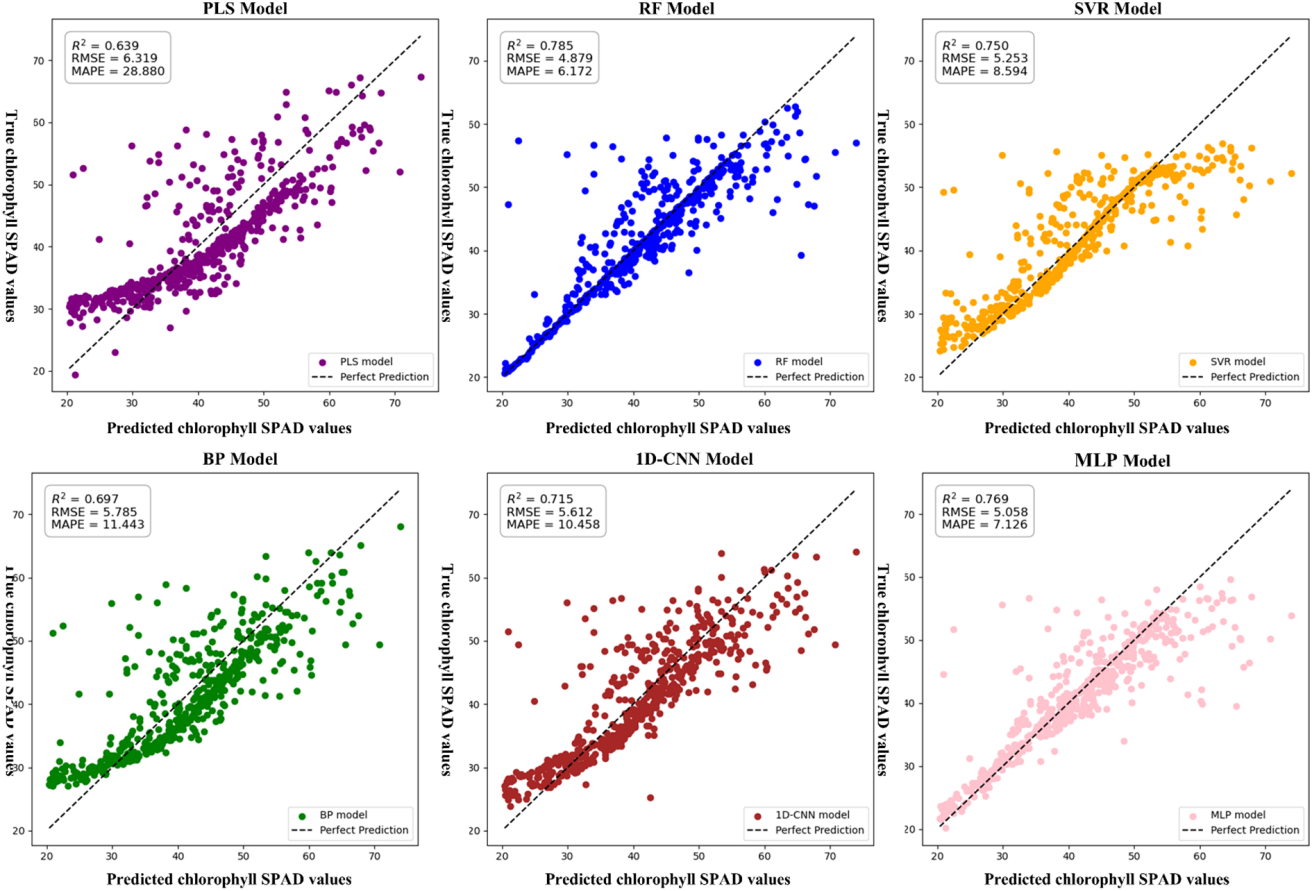
Chlorophyll estimation model based on texture features only.SPAD: A unitless index derived from SPAD meter readings, commonly used to estimate the relative chlorophyll concentration in plant leaves.

In the texture feature–only models, RF outperformed the others. Its R² was 22.85%, 4.67%, 12.63%, 9.79%, and 2.08% higher than those of PLSR, SVR, BP, CNN, and MLP, respectively. RMSE was reduced by 29.51%, 7.67%, 18.57%, 15.02%, and 3.67%, while MAPE decreased by 367.92%, 39.24%, 85.40%, 69.44%, and 15.46%. A summary of these performance differences is presented in **Figure 8**.

**Figure 9:**
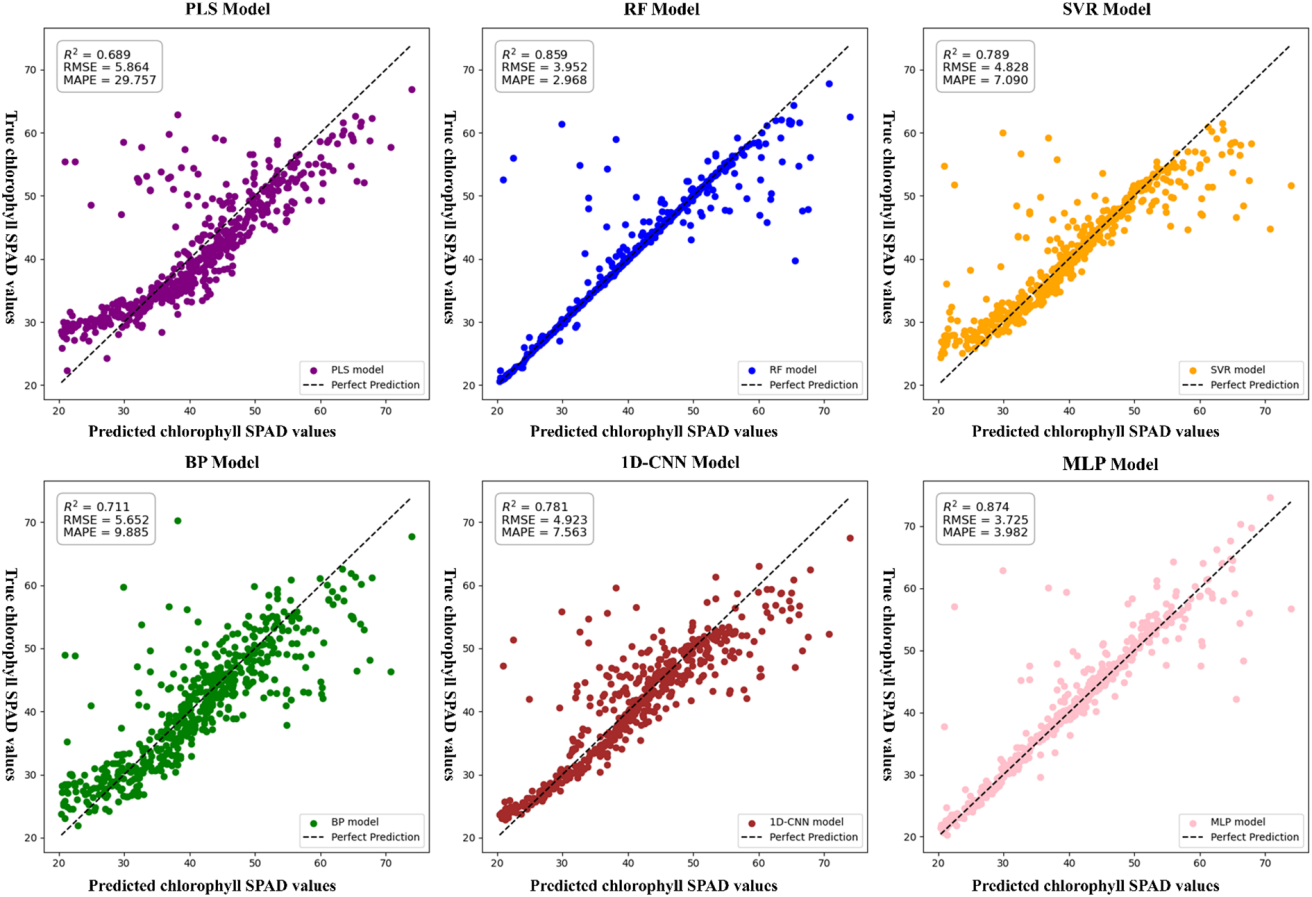
Chlorophyll estimation model incorporating vegetation indices and texture features.SPAD: A unitless index derived from SPAD meter readings, commonly used to estimate the relative chlorophyll concentration in plant leaves.

**Figure 10:**
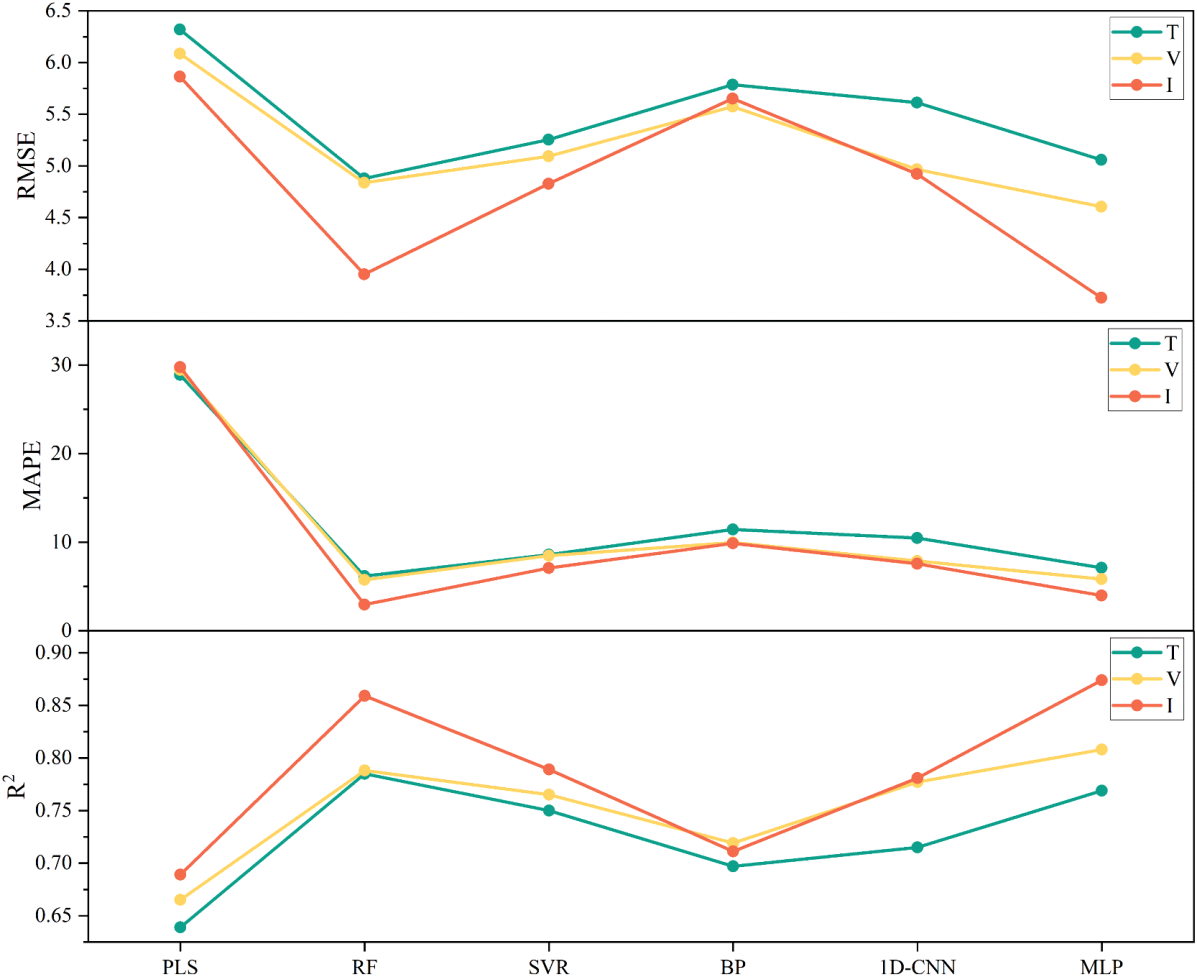
Impact of different input feature types on chlorophyll estimation model accuracy. T, Texture features; V, Vegetation indices; I, Integrated features combining both vegetation indices and texture features.

As illustrated in **Figure 9**, when vegetation index and texture features were combined as input features, the accuracy of all models improved. RF exhibited the largest improvement, with R² increased by 9.01%, RMSE decreased by 22.39%, and MAPE reduced by 93.30% compared to its vegetation index–only model. However, MLP still achieved the highest overall accuracy. These performance enhancements across models are detailed in **Figure 10**. Therefore, the MLP model based on combined vegetation index and texture features was selected for dynamic chlorophyll content monitoring in the photovoltaic area.

### 3.3 Application of the optimal monitoring model for vegetation chlorophyll estimation

**Figure 11:**
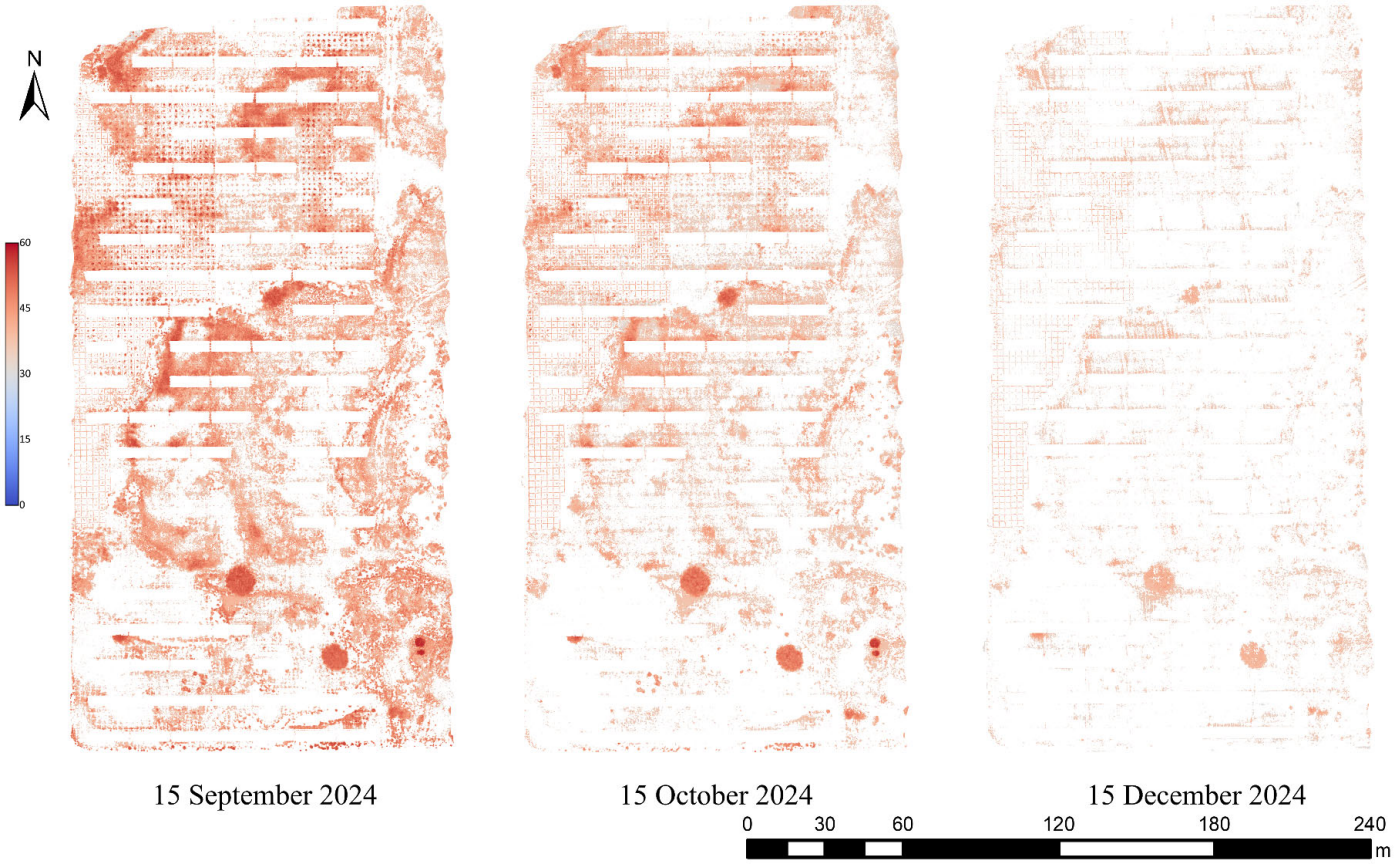
Application of the optimal MLP model for chlorophyll content estimation.

In this study, based on the single-band mosaicked images acquired from the UAV multispectral camera, a total of 10 vegetation indices and 11 texture features exhibiting strong correlations with chlorophyll content (as identified in Section 3.2) were automatically calculated. These features were spatially aligned using a sliding window approach and served as input variables for the MLP model. Specifically, the sliding window approach refers to calculating texture features within a fixed-size neighborhood centered on each pixel, allowing the local spatial characteristics to be quantified and associated with the corresponding vegetation index values at the same location. The extracted features at each pixel location were then input into the trained MLP model to generate a chlorophyll content prediction, resulting in a continuous spatial distribution map of vegetation chlorophyll across the entire study area, as shown in **Figure 11**. It is important to note that these maps were produced by applying the previously validated MLP model, ensuring that the displayed spatial patterns are based on statistically reliable predictions rather than visual interpretation alone.

The results indicate pronounced spatiotemporal heterogeneity in chlorophyll content across the experimental area. Temporally, vegetation exhibited higher chlorophyll content on September 15, whereas values were markedly lower on December 15, revealing an overall decreasing trend. Spatially, a consistent pattern of higher chlorophyll levels in the eastern region and lower levels in the western region was observed, aligning well with ground truth measurements.

In conclusion, the chlorophyll content estimation model constructed using vegetation indices and texture features extracted from UAV multispectral imagery, combined with a MLP model, provides reliable chlorophyll distribution estimates across space and time. This approach offers valuable data support for precision vegetation management and the safe operation of photovoltaic power stations.

## 4 Discussion

In this study, a UAV-mounted multispectral camera equipped with four spectral bands—green, red, red-edge, and NIR—along with a high-definition digital camera, was used to efficiently acquire vegetation growth information. Compared with UAV hyperspectral sensors, satellite platforms, and large-scale aerial remote sensing, UAV multispectral remote sensing offers advantages such as easier data processing, lower cost, and reduced sensitivity to atmospheric conditions like cloud cover, making it suitable for fine-scale vegetation monitoring (Qiao et al., 2022).

Chlorophyll, a key pigment required for photosynthesis, significantly influences the nitrogen content and photosynthetic capacity of vegetation, serving as a critical indicator of plant health and growth status. Previous studies have demonstrated the effectiveness of UAV multispectral remote sensing in monitoring chlorophyll content in economic crops such as oilseed rape (Lukas et al., 2022), wheat (Cui et al., 2019), and tobacco (Khan et al., 2020). For instance, Lang et al. segmented maize canopy images from the soil background based on UAV RGB imagery and constructed a chlorophyll content estimation model using a back-propagation neural network. The model achieved an R² of 0.7246 and an RMSE of 4.4425 (Lang et al., 2019). However, their study relied solely on visible bands, lacking NIR and red-edge information, which are crucial for capturing vegetation biochemical characteristics, particularly chlorophyll content. In contrast, the present study utilized vegetation indices derived from multispectral bands and incorporated texture features extracted from these spectral channels, resulting in improved model performance (R² = 0.874, RMSE = 3.725). Moreover, their study demonstrated an 8.26% increase in R² when incorporating texture features, which closely aligns with the 9.01% improvement observed in our work. Yang et al. employed four machine learning algorithms to estimate potato chlorophyll content across different growth stages, using six features including vegetation indices and texture features. Among the models, the Stacking algorithm yielded the best performance, with R² and RMSE values of 0.739 and 0.511, respectively (Yang et al., 2022). Compared with their approach, the present study incorporated three deep learning algorithms—BPNN, 1D-CNN, and MLP—and achieved higher predictive accuracy. Furthermore, their findings also demonstrated the positive contribution of texture features to chlorophyll estimation accuracy. Zhao et al. employed drone-captured multispectral remote sensing imagery in combination with random forest, gradient boosting, and regularized regression algorithms to develop predictive models for three key rice plant parameters: leaf water content, chlorophyll content, and leaf area index. Their analysis revealed that while the random forest algorithm demonstrated superior predictive accuracy for both leaf water content and chlorophyll content models, gradient boosting and regularized regression techniques exhibited better performance for leaf area index modeling (Zhao et al., 2025). This finding highlights the strong performance of RF for chlorophyll estimation, which is consistent with the results of the present study, where the RF model achieved the best performance among texture-only models. While the R² obtained in our study was slightly lower, this discrepancy may be attributed to the influence of photovoltaic panel shading, which can reduce the accuracy of multispectral reflectance values used in model training.

However, the application of UAV multispectral remote sensing in arid regions, particularly for vegetation monitoring in photovoltaic power stations, remains underexplored. Thus, this study references existing research on both economic crops and arid-region vegetation for vegetation index selection, and integrates multispectral texture features to evaluate the performance of various models in photovoltaic vegetation monitoring (Ma et al., 2023).

Initially, the correlations between vegetation indices, texture features, and chlorophyll content were analyzed. The results were consistent with prior studies, revealing significant correlations. Although Pearson correlation analysis revealed multicollinearity among certain vegetation indices and texture features, these variables also ranked highly in the mRMR feature selection process. Since mRMR prioritizes relevance to the target variable while minimizing redundancy, some degree of inter-feature correlation was tolerated to preserve important predictors. Moreover, among the six models used in this study, PLSR and RF are inherently robust to multicollinearity, further justifying the inclusion of these correlated features. This trade-off reflects a deliberate balance between reducing redundancy and maintaining valuable information for model training. The vegetation index GNDVI exhibited the strongest correlation (Pearson’s r = 0.82), followed by the texture feature NIR_Mean (r = 0.65). Notably, these correlations were slightly lower (by 0.05–0.10) than those reported for economic crops. Zhu et al. indicated that drought stress can reduce the correlation between vegetation indices and chlorophyll content, which aligns with the sandy experimental area’s characteristics of low water retention and high evapotranspiration (Zhu et al., 2024). The influence of environmental factors on chlorophyll correlation will be further investigated in future studies.

Some texture features excluded during selection did not exhibit strong correlations with chlorophyll content. Given that multispectral texture features were extracted based on a sliding window and directional parameters, this study set the extraction window to 8×8 pixels and the calculation direction to 90°, referencing practices from agricultural research. Due to the photovoltaic area’s complex vegetation and topography, future research should explore adaptive window sizes and computational angles to enhance the accuracy of texture feature extraction.

Additionally, the differential performance of models observed in this study underscores the importance of matching model structures to input data characteristics. MLP’s superior performance with vegetation indices and combined features highlights its effectiveness in capturing complex nonlinear patterns, whereas RF’s advantage with texture features reflects its strength in handling heterogeneous, high-dimensional data. This insight can guide future model selection and optimization for UAV-based vegetation monitoring applications.

As shown in **Figure 11**, the chlorophyll content exhibited a distinct spatial and temporal pattern across the study area, with generally higher values observed in the eastern region compared to the west, and a noticeable decline from September to December. Field observations indicated that vegetation in the eastern portion of the site showed better growth and higher canopy coverage, which may be attributed to microtopographic conditions. The western area is generally at a higher elevation, potentially reducing soil moisture retention and limiting vegetation development. In addition, the western region is more open and exposed to wind and sand, which can further inhibit plant growth. Seasonally, the observed decline in chlorophyll content from September to December aligns with decreasing temperatures and shortened daylight duration during the autumn and early winter months. These environmental conditions reduce photosynthetic activity and chlorophyll synthesis, resulting in lower chlorophyll content in the December measurements. Although the prediction map for December 15 showed a less pronounced spatial gradient, this likely reflects reduced physiological activity and overall chlorophyll variability during the winter season, rather than a limitation of the model. Importantly, these predictions were generated using a previously validated MLP model and reflected spatial distributions that aligned with field observations, even under low-variability seasonal conditions. On the whole, these spatial and temporal patterns in chlorophyll content appear to be predominantly driven by natural environmental factors, including topography, climate, and seasonal plant senescence.

Moreover, vegetation growth appeared more robust between photovoltaic panels, possibly due to reduced transpiration caused by panel shading. However, this shading effect also introduced local anomalies in multispectral reflectance, especially in shaded areas where light intensity was significantly reduced, thereby limiting the model’s ability to accurately estimate chlorophyll content. To mitigate this issue, UAV data acquisition was conducted around solar noon to minimize the extent of shadowed areas. Seasonal variations in solar radiation intensity also affected spectral reflectance, particularly due to differences in sunlight angle and duration across acquisition dates. Although radiometric calibration plates (25% and 50% reflectance) were photographed prior to each flight to reduce the effects of changing illumination conditions, environmental differences across time periods may still have contributed to model uncertainty. In addition, all models were trained and validated using data collected from fixed locations and specific time points. Therefore, the generalizability of the method to other geographic areas or different temporal conditions has yet to be verified. In response to these limitations, future work will incorporate oblique multispectral imagery to better capture vegetation shaded by photovoltaic panels from multiple angles, enabling the extraction of complementary variables from orthomosaic images. Combining nadir and oblique images will facilitate more accurate reconstruction of three-dimensional models for structural analysis, ultimately enhancing the robustness and applicability of chlorophyll estimation methods.

In addition, we plan to explore the application of UAV-based hyperspectral imagery, which offers finer spectral resolution and greater spectral richness compared to multispectral data, with the aim of improving model performance in estimating chlorophyll and other vegetation parameters. Variables derived from digital surface models or digital terrain models, either generated using Structure-from-Motion techniques or created directly from lidar point clouds, will also be considered to account for topographic or structural influences. Integrating these diverse data sources—including spectral, structural, and topographic information—is expected to enhance model generalizability and provide a more comprehensive understanding of vegetation characteristics.

Furthermore, to improve generalizability and minimize the influence of confounding factors such as environmental variability and sensor differences, future studies are recommended to adopt standardized data acquisition protocols and consider alternative experimental designs, such as randomized block designs or stratified sampling schemes. These approaches can better account for spatial and temporal heterogeneity and improve the robustness of model performance. These findings provide not only a technical basis for chlorophyll estimation in photovoltaic areas but also practical guidance for UAV-based vegetation monitoring in regions where ground-based sampling is limited or unavailable. The proposed modeling framework can be adapted for similar crop types or arid environments by adjusting input variables and model parameters. In the absence of ground truth data, researchers may use models that were trained in similar environments. These models can be adjusted using transfer learning techniques and verified with indirect indicators, such as canopy cover or growth stages, which may provide a practical pathway to achieve reliable chlorophyll estimation.

Moreover, to further improve the model’s generalizability and assess its transferability across varying environmental and management conditions, additional studies in different geographic regions with the same crop species are strongly recommended. Such efforts would help evaluate how well the trained models perform beyond the original study area and identify any necessary adaptations, thereby strengthening the reliability and applicability of UAV-based chlorophyll prediction in broader contexts.

In conclusion, the fusion of vegetation indices and texture features as model inputs significantly enhanced chlorophyll estimation performance. Furthermore, deep learning regression algorithms provided higher predictive accuracy compared to results reported in previous studies. Although photovoltaic panel shading and other environmental factors may introduce localized uncertainties and reduce model accuracy, the approach still effectively supports fine-scale monitoring of vegetation chlorophyll content in photovoltaic power plant areas. This provides a valuable foundation for ensuring the safe and stable operation of photovoltaic systems.

## 5 Conclusions

Quantitative relationships were established between vegetation indices and texture features extracted from UAV multispectral remote sensing data and the chlorophyll content of ground-measured vegetation across different time periods. Three feature input strategies were employed: using vegetation indices only, texture features only, and a combination of both. Based on these inputs, six regression algorithms—PLSR, RF, SVR, BPNN, CNN, and MLP—were utilized to construct inverse models for estimating vegetation chlorophyll content in photovoltaic power plant areas The optimal number of vegetation index features for constructing the fitted model was 10, while the number of texture features was 11. Among them, the vegetation index with the highest Pearson correlation coefficient with chlorophyll content was GNDVI (p = 0.82), and the texture feature was NIR_Mean (p =0.65). The chlorophyll fitting model constructed using only texture features exhibited the lowest accuracy, while the model based solely on vegetation indices demonstrated slightly better performance. In contrast, the model incorporating both vegetation indices and texture features showed improved accuracy compared to either feature set alone. Notably, the RF model achieved the greatest improvement, with a 9.01% increase in R², a 22.39% decrease in RMSE, and a 93.30% reduction in MAPE. The MLP-based model integrating vegetation indices and texture features achieved the highest accuracy, with R² of 0.874, RMSE of 3.725, and MAPE of 3.982. This level of accuracy satisfies the requirements for refined vegetation monitoring in photovoltaic power plant areas and provides reliable information support for scientific and safe production management. Further research on improving model generalizability and incorporating additional variables, such as those derived from oblique UAV imagery or topographic data, could help enhance the accuracy and robustness of chlorophyll content estimation.

## Data Availability

The raw data supporting the conclusions of this article will be made available by the authors, without undue reservation.
